# Marginal costing methods highlight the contributing cost of comorbid conditions in Medicare patients: a quasi-experimental case–control study of ischemic stroke costs

**DOI:** 10.1186/1478-7547-11-29

**Published:** 2013-11-16

**Authors:** Annie N Simpson, Heather S Bonilha, Abby S Kazley, James S Zoller, Charles Ellis

**Affiliations:** 1 Department of Healthcare Leadership and Management, Medical University of South Carolina, 151 Rutledge Avenue, MSC 962, Charleston, SC 29425, USA; 2 Department of Health Sciences and Research, Medical University of South Carolina, 151 Rutledge Avenue, MSC 962, Charleston, SC 29425, USA; 3 Department of Otolaryngology-Head and Neck Surgery, College of Medicine, Medical University of South Carolina, 151 Rutledge Avenue, MSC 962, Charleston, SC 29425, USA

**Keywords:** Marginal cost, Attributable cost, Methods, Rehabilitation, Ischemic stroke

## Abstract

**Background:**

Cost of illness studies are needed to provide estimates for input into cost-effectiveness studies and as information drivers to resource allocation. However, these studies often do not differentiate costs associated with the disease of interest and costs of co-morbidities. The goal of this study was to identify the 1-year cost of ischemic stroke compared to the annual cost of care for a comparable non-stroke group of South Carolina (SC) Medicare beneficiaries resulting in a marginal cost estimate.

**Methods:**

SC data for 2004 and 2005 were used to estimate the mean 12 month cost of stroke for 2,976 Medicare beneficiaries hospitalized for Ischemic Stroke in 2004. Using nearest neighbor propensity score matching, a control group of non-stroke beneficiaries were matched on age, gender, race, risk factors, and Charlson comorbidity index and their costs were calculated. Marginal cost attributable to ischemic stroke was calculated as the difference between these two adjusted cost estimates.

**Results:**

The total cost estimated for SC stroke patients for 1 year (2004) was $81.3 million. The cost for the matched comparison group without stroke was $54.4 million. Thus, the 2004 marginal costs to Medicare due to Ischemic stroke in SC are estimated to be $26.9 million.

**Conclusions:**

Accurate estimates of cost of care for conditions, such as stroke, that are common in older patients with a high rate of comorbid conditions require the use of a marginal costing approach. Over estimation of cost of care for stroke may lead to prediction of larger savings than realizable from important stroke treatment and prevention programs, which may damage the credibility of program advocates, and jeopardize long term funding support. Additionally, correct cost estimates are needed as inputs for valid cost-effectiveness studies. Thus, it is important to use marginal costing for stroke, especially with the increasing public focus on evidence-based economic decision making to be expected with healthcare reform.

## Background

The economic burden of illness for a disease, such as acute ischemic stroke (AIS) is usually reported as direct total healthcare cost incurred over time, and these costs are only rarely compared to the cost of care for an equally ill, non-stroke, control group. Therefore the outcome of cost of illness studies in stroke often misrepresent costs in a population that tends to have a great amount of care costs that are related to comorbidities. The reported costs of stroke are high, so understanding the difference between the cost of the stroke and the cost of care for comorbid conditions is important for fiscal planning, cost-effectiveness studies, and policy assessment purposes.

The economic burden of stroke is potentially substantial, since the estimates are likely inflated. Estimates indicated that in the US stroke related costs reached 73.7 billion dollars in the year 2010 alone [1]. In the state of South Carolina (SC) which is known for some of the highest stroke rates in the US, hospitalization costs from stroke were estimated at $499 million in 2008 [2] with additional indirect costs due to loss of wages, productivity and pre-stroke social roles. Stroke in SC and the remainder of the US is expected to be exacerbated by the aging population of baby boomers.

Ischemic stroke accounts for 87% of all strokes, with the remaining strokes falling into one of the hemorrhagic categories [1,3]. Interventions to treat acute ischemic stroke are increasingly being used which could effectively reduce mortality rates, however, long-term morbidity due to stroke-related impairments are expected to increase.

Despite Medicare’s attempts to contain costs, the total cost of stroke and other chronic illness in the US continues to rise. Some speculate that the cost of treatment after ischemic stroke will increase as mortality rates decrease and stroke survivors live longer after stroke with their stroke-related disabilities [4]. Such speculation is difficult to confirm or deny because of the significant variability in cost of illness research models and methods. However, because stroke-related interventions are likely to facilitate longer lifespans among those experiencing a stroke and there has been an increase in the comorbidity in the elderly population, an understanding of the economics of stroke is becoming increasingly important. Specifically, understanding the marginal cost of stroke, i.e. those costs over and above normal expected medical care, can serve as a valuable cost benchmark that can be followed accurately over time. Furthermore, it is important to know what proportion of stroke health services is attributable to the initial hospital admission and rehabilitation, and what proportion are actually attributable to comorbidities. Therefore the purpose of this study was to examine the 1-year cost of ischemic stroke, cost of hospital admission, and rehabilitation services and compare those annual costs of care to a comparable non-stroke group of South Carolina (SC) Medicare beneficiaries. It is expected that the analysis will result in a marginal cost estimate of stroke itself.

## Methods

This research project used marginal cost estimation, also known as case–control cost comparison, to estimate cost of care for patients with acute ischemic stroke. Marginal cost was calculated by estimating the costs of resources used for care after an index event, using population based patient-level data, less the cost of a similar patient population who do not have the illness, over the same period of time. Arguably, this is one of the more precise ways to estimate overall cost-of-illness, especially in the case of stroke where most patients are older and have many comorbid conditions which tend to increase cost [5].

### Data

A retrospective longitudinal cohort of patients with a primary diagnosis of ischemic stroke was extracted from the SC Medicare hospital discharge Standard Analytic File (SAF) database that contained data for the years 2003, 2004, and 2005. Medicare is a government funded health insurance program developed to cover individuals over age 65. Medicare may also insure individuals under 65 who are disabled or anyone who is diagnosed with end stage renal disease. All Americans over 65 years of age are automatically enrolled in Part A Medicare coverage that provides insurance for hospitalizations at no cost to the enrollee. Other parts of Medicare coverage are also available but may require monthly premiums. Beneficiaries’ data were linked from different claims files via encrypted beneficiary identification numbers at the individual level and followed longitudinally in two groups.

The stroke and the control cohort were then followed for the remaining time period of available data up until either death or 365 days from index date. Where data was available in the six months prior to the index date for each cohort, diagnosis coding was used to construct the Charlson comorbidity Index up until and including index stroke for each patient. The data used in this study were provided by the SC Office of Research Services (ORS) from a state-wide cohort of Medicare participants from SC which was made available to the researchers as part of the EXCEED grant (South Carolina EXCEED Project funded by AHRQ under DUA #16339 EDG#4081) to examine health disparities in minority populations.

### Patient selection, settings and exclusions

Patients were selected based on the presence of a primary diagnosis of AIS via the most consistently accurate and highly specific ICD-9-CM codes of 434.xx or 436.xx in the hospital impatient claims file [6]. Patients were excluded if their index stroke date did not take place in 2004 or if they had a stroke admission in the six months prior to the index admission (n = 152 excluded due to previous stroke in prior 6 months), where data were available. This was done to try to exclude strokes in our indexed stroke count that were recent re-stroke admissions. Any patients less than 65 years of age at the time of the hospital admission for their index stroke were also excluded because such patients represent a different subpopulation than traditional Medicare beneficiaries (n = 575 excluded due to age < 65). Patients with missing race information were also excluded because race is important to stroke-related and economic outcomes (n = 22 due to missing race information). A cohort of 2,679 Medicare patients with an ischemic stroke in 2004 were identified and included in the analysis. A control group was selected using propensity score matching for comparison from a group of 17,924 potential Medicare beneficiaries using the same inclusion criteria with the exclusion of stroke.

### Propensity score matching to control group

Two controls were selected for each stroke case using a matching scheme based on propensity score (PS) similarity. PS methods using a greedy-match algorithm with caliper distance set to a width of 0.1 standard deviations of the logit of the estimated propensity score and a 1 case to 2 control matching scheme, without replacement, was used to match cases with controls in the final analytical data set. Known components related to the risks of having a stroke were included as covariates in the logistic regression model [7]. Covariates used in the PS model included age, gender, race, Charlson comorbidity score, and the following stroke risk factors (as monitored in billing records from the six months prior to index study start date): diabetes, heart failure, heart attack, atrial fibrillation, and hypertension as well as five interaction terms of gender by each risk factor. Final model selection was based on hypothesis testing of balance between groups of all the covariates included in the propensity score logistic regression models, model with largest r-square value, and satisfied reduction of the absolute standardized differences in means between the matched and unmatched data for all covariates (<0.25).

### Statistical analysis

The marginal cost of stroke-related healthcare and stroke-related rehabilitation care was defined as the cost of caring for patients with stroke after index stroke hospital admission that is over and above the cost of general medical care in the non-stroke control group over the same time period. Total Medicare payments and total rehabilitation services payments were calculated using means and unadjusted differences between groups were tested using non-parametric Wilcoxon Scores. The marginal cost of stroke-related rehabilitation was calculated by subtracting the average total rehabilitation services payments for the controls, from the average total rehabilitation services payments for the stroke cases. Rehabilitation services were selected based on secondary diagnoses using a series of ICD-9-CM codes (not shown).

Generalized linear modeling techniques were used to estimate average annual total Medicare payments for stroke cases and controls and to test for differences between the stroke case and control groups. The 1-year marginal cost of stroke-related healthcare was calculated by subtracting the estimated total Medicare payments for the controls from the estimated total Medicare payments for the stroke cases.

To correct for the non-normal distribution of Medicare costs, gamma distributed generalized linear models using a logarithmic transformation [8] were analyzed using the PROC GENMOD module in the SAS statistical software. The use of a gamma distributed generalized linear model with a log transformed link function has been shown to be a good method to estimate healthcare cost distributions that are generally right-skewed, especially when the log transformed dependent variables do not have heavy tails or excessive heteroscedasticity such as was found to be true in these data [9]. This type of model was found to fit these study data well.

Multicollinearity between covariates was assessed using Pearson correlation coefficients. No collinearity was found in these data (all p-values > 0.25). Clinically relevant variables were used to determine which covariates were initially included in the models to control for population differences. Covariate adjustment was used to control for differences in cost that may be attributed to other factors. Covariates that were tested for potential confounding in model estimates of stroke and rehabilitation payments included age, gender, race, Charlson morbidity score, and number of days alive in the year after index date. Manual backwards selection regression methods were used to decide which covariates remained in the final models using smallest Akaike Information Criterion (AIC), Bayesian Information Criterion (BIC) values, change in parameter estimates, and covariate p-values to judge model fit. Variables with p-values greater than 0.20 were removed from the models if the AIC and BIC values became smaller than in the previous model containing the covariate. All analyses were performed using SAS statistical software (version 9.2; SAS Institute, Inc., Cary, NC), and statistical significance was determined at the 0.05 level. The Medical University of South Carolina Institutional Review Board approved the study proposal.

## Results

Each of the propensity score matched covariates resulted in statistically equal groups on known potentially biasing factors (Table 1). Unadjusted outcomes such as, proportion receiving any rehabilitation services in the year after index date and proportion who died in the year after index study date, were higher in the stroke cohort versus the control group (p-values <0.0001) (Table 1). The average number of days alive in the year after index study start date (stroke date for the stroke cohort and randomly selected 2004 medical bill date for the control group) were higher in the control group than the stroke group (p-value < 0.0001) (Table 1).

**Table 1:** Demographics and Characteristics of 2004 ischemic stroke patients and Matched controls

	**Overall**	**2004 Stroke**	**2004 Controls**	
	**(n = 8928)**	**(n = 2976)**	**(n = 5952)**	p-value*
Age (Approximate)^†^	78.2 (±6.8)	78.1 (±6.9)	78.3 (±6.8)	0.14
Male^	3510 (39.3)	1149 (38.6)	2361 (39.7)	0.33
Caucasian^	6374 (71.4)	2148 (72.2)	4226 (71.0)	0.24
Charlson morbidity score^†^	2.1 (±1.7)	2.1 (±1.7)	2.0 (±1.6)	0.10
Receiving rehabilitation^	2438 (27.3)	1303 (43.8)	1135 (19.1)	<0.0001
Died year post-index date^	1975 (22.1)	967 (32.5)	1008 (16.9)	<0.0001
Days alive yr post-index date^†^	309.1 (±115.2)	279.2 (±136.1)	324.0 (±99.8)	<0.0001
Stroke risk factors (prior 6 months)				
Diabetes^	3018 (33.8)	991 (33.3)	2027 (34.1)	0.48
Heart failure^	2606 (29.2)	851 (28.6)	1755 (29.5)	0.38
Heart attack^	767 (8.6)	280 (9.4)	487 (8.2)	0.06
Atrial fibrillation^	2423 (27.1)	832 (28.0)	1591 (26.7)	0.22
Hypertension^	7512 (84.1)	2476 (83.2)	5036 (84.6)	0.09

^†^Mean (±SD).

^^^N (%).

*p-values were calculated using non-parametric Wilcoxon Scores for continuous measures, Chi-square or Fisher’s Exact for categorical measures (as appropriate).

**Figure 1:**
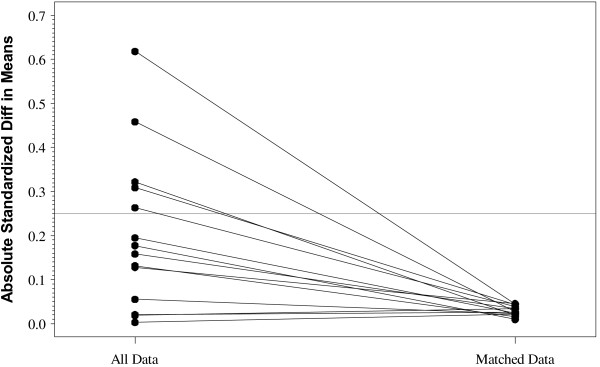
Standardized Difference of Means of PS Covariates Before and After Matching.

A plot of the absolute standardized differences in means (Figure 1) offers a good representation of whether selection bias of known factors has been reduced by matching on propensity score [10]. Balance (i.e. reduction in standardized mean differences between stroke cases and selected controls) for each covariate that was used in the logistic regression model of propensity is improved when differences are reduced after matching to no greater than 0.25 [11]. Figure 1 illustrates that the standardized differences in means for each of the fourteen covariates included in the propensity model is reduced to less than the 0.25 recommended level after matching, indicating good control of potential selection bias of known confounders.

### Marginal cost of stroke

The estimated average per beneficiary healthcare cost in the first year after ischemic stroke in 2004 was $27,330 (Table 2). Average estimated healthcare costs in the control group was significantly lower at $18,276 (p-value <0.0001) (Table 2). Therefore, the marginal cost of stroke, defined as the cost of healthcare for an average individual who has had an ischemic stroke, over and above the normal average cost seen in a similar group who have not suffered a stroke, was the difference between these group estimates, or $9,054 (Table 2).

**Table 2:** **Rehabilitation services Medicare costs for 2004 stroke cohort and Matched controls**^**†**^

	**Overall**	**2004 Stroke**	**2004 Controls**	
	**(n = 8928)**	**(n = 5952)**	**(n = 2976)**	p-value*
Total Medicare payments	21,220 (28267)	27,329 (23913)	18,165 (29745)	<0.0001
Estimated total Medicare payments^#^		27,330	18,276	<0.0001
1-year marginal cost of stroke services		$9,054		
Total rehabilitation services payments	1,968 (5887)	3,735 (8245)	1,084 (3958)	<0.0001
1-year marginal cost of stroke rehabilitation services		$2,651		

^†^All payments are in 2004 US$ and are reported as mean (SD); payments have been rounded to the nearest dollar.

*p-values for univariate comparisons were calculated using non-parametric Wilcoxon Scores for continuous measures.

^#^p-values for estimated payments were calculated using multivariable Log linked Gamma Distributed Generalized Linear Model.

**Figure 2:**
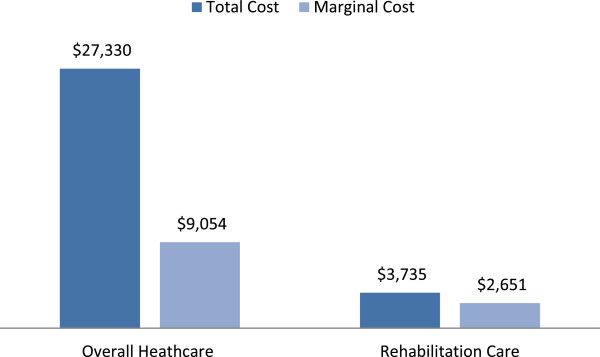
One Year Cost of Ischemic Stroke in SC (2004).

The average cost of rehabilitation after stroke in 2004 was $3,735, significantly more than average rehabilitation services cost for the controls (p-value <0.0001) (Table 2). The difference between the rehabilitation costs in these two groups is the 1-year marginal cost of stroke-related rehabilitation care, or $2,651 (Table 2). Both the average total healthcare cost differences and rehabilitation cost differences are illustrated in Figure 2.

Based on stroke estimates from the current study, 1-year total healthcare cost to SC Medicare in 2004 of beneficiaries having ischemic stroke was $81,334,080 and total annualized 2004 SC Medicare rehabilitation costs due to ischemic stroke was $11,115,360 (Table 3). However, when considering marginal rather than total cost, the annualized stroke-related healthcare costs to SC Medicare in 2004 were $26,944,704 (Table 3). This reflects a potential $54.4 million over-estimation of Medicare expenditures that were not due to ischemic stroke. Similarly, the marginal cost of stroke-related rehabilitation care in 2004 in SC Medicare patients having ischemic stroke was $7,889,376, resulting in a $3.2 million over-estimation if total rehabilitation costs are quoted (Table 3).

**Table 3:** Cost of ischemic stroke to Medicare in SC and the US

	**2004 SC Stroke costs**^**#**^	**2012 Projected SC stroke costs**^**†**^	**2012 Projected US stroke costs**^**†**^	**2004 SC Rehab-related stroke costs**^**#**^	**2012 Projected SC Rehab-Related stroke costs**^**†**^	**2012 Projected US Rehab-Related stroke costs**^**†**^
Total cost	$81.3 M	$109.5 M	$7,319.3 M	$11.1 M	$15.0 M	$1,000.3 M
Marginal cost	$26.9 M	$36.3 M	$2,424.8 M	$7.9 M	$10.6 M	$710.0 M
Differential	$54.4 M	$73.2 M	$4,894.5 M	$3.2 M	$4.4 M	$290.0 M

*Cost estimates are based on costs and incidence rates using the 2004 Stroke Group from this study and are rounded to the nearest tenth of a million $US dollar.

#The number of 2004 Medicare beneficiaries used to estimate SC costs to Medicare was taken from Kaiser Family Foundation State Health Facts website (http://www.statehealthfacts.org, accessed on 05/25/2012).

†2012 $US dollars cost projections were calculated using 2004 estimates inflated by Consumer Price Index series CUUR0000SAM2 annual medical care services adjustment (Bureau of Labor Statistics).

When these 2004 SC Medicare stroke cost estimates are inflated using the Consumer Price Index for healthcare services to make 2012 dollar projections, 2012 SC Medicare annualized stroke-related healthcare and rehabilitation care costs would be over-estimated by $73.2 million and $4.34 million, respectively [12] (Table 3). When SC stroke rates and costs estimated in this study are used to predict Medicare costs for all US ischemic stroke patients, 2012 total stroke costs are predicted to be $7.32 billion compared with 2012 marginal stroke-related healthcare costs of only $2.42 billion, resulting in an over-estimation of stroke-related expenditures of $4.89 billion (Table 3). Similarly, utilizing total cost instead of marginal cost figures to predict stroke-related rehabilitation care costs of 2012 Medicare beneficiaries would result in an over-estimate of $290 million (Table 3).

## Discussion

The one-year healthcare costs attributable to ischemic stroke was examined by calculating the marginal estimated healthcare Medicare payment difference between a 2004 ischemic stroke cohort and a propensity matched 2004 non-stroke control group. The estimated marginal 2004 cost of stroke-related healthcare in the year after ischemic stroke in SC was $9,054. This amount reflects the difference between the annualized healthcare cost of the stroke group of $27,330 less the healthcare costs of the propensity score matched non-stroke control group of $18,276 (p-value <0.0001). Similarly, the 2004 marginal cost of stroke-related rehabilitation services in the year after ischemic stroke was $2,651, resulting from a statistically significant difference between the average annualized total rehabilitation services cost for the stroke group of $3,735 and the control group of $1,064 (p-value <0.0001). The findings clearly demonstrated that the total cost of healthcare after ischemic stroke significantly overestimates the cost of stroke as an illness because the marginal cost of stroke in this older population is attenuated by healthcare costs related to comorbidities.

While these amounts are substantial and statistically significant, they are lower than estimates commonly reported. The most frequently cited estimates are by Taylor, Davis and colleagues (cited in 559 publications referencing this research (accessed May 2012)) [13]. Interestingly, the Taylor study continues to be referenced in the “Heart Disease and Stroke Statistics—annual update: A report from the American Heart Association” [14-18], even though this research is based on 1990 data and does not benefit from recent improvements to methodology. This continued referencing is likely the result of no new estimates emerging in the literature using population-based studies on the cost of stroke.

Further, the authors of the Taylor study estimated the 1990 annual direct cost of ischemic stroke in the 65–74 year age group to be $17,823 ($35,197 in 2004 dollars) versus the annual healthcare expenditures for their control group of $2,825 ($5,579 in 2004 dollars) [13]. The Taylor study reports the annual direct marginal cost of ischemic stroke of $14,998 in 1990 dollars ($29,618 in 2004 inflated dollars) which is much higher than we found in the current research. Differences between the current research and that undertaken by Taylor and colleagues in their 1997 publication are likely related to differences in the methods used to estimate these dollar amounts and constantly changing healthcare practice patterns.

A significant trend toward shorter hospital average length of stay has been frequently reported in the literature which is also indicative of changing practice patterns and third-party payer reimbursement changes over time [19]. For example, the Taylor study reports that 70% of the medical costs for the first year after a stroke can be accounted for by the initial hospitalization [13]; however, the article does not report the average length of stay. In the current study, initial hospital costs only account for 35% of the first year of total healthcare costs. This may reflect the declining length of hospital stays [19-21]. Shorter length of stay would result in lower first year healthcare cost estimates in later studies, however these reductions may be counteracted by inflation adjustment over time.

The Taylor et. al. study includes a 5% Medicare sample of the 1990 US population, while the current study uses all SC Medicare patients with ischemic strokes in 2004. Taylor et. al. also used an average cost to charge ratio for all admissions to estimate stroke costs. Estimates based on cost to charge ratios for all admissions can skew costs because stroke costs may not follow general hospital cost to charge trends. The current research uses actual payments made by Medicare to the provider and does not make any assumptions in the costing methods. Also, the Taylor research used a 1 in 1,000 randomly selected, non-matched control group from the general US Medicare population. This practice would likely under estimate the control groups’ healthcare expenditures because the average Medicare population is more likely to be healthier than a control group matched on an equally at-risk population. This type of matching approach would result in inflated marginal cost estimates. The current study addresses the potential selection bias caused by unmatched controls by using propensity score matching to match the stroke cases to controls, which results in a more conservative and reasonable marginal cost estimate. An added benefit of the matching approach used in the current study was a reduction in the selection bias of other unmeasured factors that are correlated with the known covariates used in this matching algorithm. This gives confidence that the observational study is well matched and is unlikely to contain much selection bias.

In a seminal study by Samsa and colleagues, the 2-year cost and survival after cerebral infarction was estimated based on 1991 data [21]. This publication, which has been cited 130 times (accessed May 2012), reports the first year cost of first ever ischemic stroke in the over 65 Medicare population as $29,444 (in 1991 dollars) [22]. The Samsa estimate is very similar to the current study’s 1-year cost estimate of $27,330, but the current study is based on 2004 Medicare payments while theirs is calculated using 1991 data. The Samsa study estimated total average cost rather than marginal cost. In 1991 the average length of hospital stay after acute stroke was considerably longer than it was in 2004 [23], resulting in inflated estimates when compared to current practice. Their estimates also used cost to charge ratio adjusted Medicare charges for all facility bills, rather than payments, which has an unknown effect on the costs incurred by Medicare. Thus, the estimates provided by Samsa et. al. are no longer applicable to the current healthcare system and do not take into account the cost of the non-stroke care that surviving stroke patients are likely to incur even if they had not had a stroke. However, the Samsa study provides valuable insight to the inflated estimation of costs that result from reporting total healthcare cost instead of marginal cost of care, particularly in the group examined in the current study. That is, older individuals tend to have significant healthcare costs outside of the particular major illness being studied, which make it essential to take into account other costs when estimating the costs attributable to a single disease.

There are many policy implications related to the use of inflated estimates for stroke. It is generally believed that commonly quoted figures related to incidence rates, prevalence rates, proportional estimates of certain services and cost estimates are inflated. However, the public, including those in the research community, continue to use and report those same numbers. This practice may negatively affect the allocation of funding for other important research since there are limited dollars to support health-related research efforts. Furthermore, when advocacy and governmental groups quote these inflated estimates they lose credibility with the public and their ability to make good policy decisions are diminished. Also, given the climate of cost-containment in health care, the accuracy of estimates of total cost of care related to any particular condition can help providers and funding groups better plan for resources to provide such care.

### Limitations

There are several limitations in the analysis of SC Medicare claims data. There is an inherent limitation of using administrative claims to ascertain diagnoses and identify resource use and costs with complete accuracy, because these data are not purposely collected for clinical research but are collected for the distinct purpose of making healthcare payments. It is possible that signs or symptoms related to stroke may not have been captured in the claims, and that ischemic stroke patients not diagnosed with 434.xx and 436.xx ICD-9-CM codes under the primary diagnosis category would be missed by this analysis. Similarly, the measurement of rehabilitation and general resource use in these data depends on the design and implementation of the Medicare fee-for-service plan and its scope of coverage which may change over time. Medicare reimbursement rules and healthcare practice patterns that change over time may also make these 2004 based results less generalizable to current times. The use of SC estimates from this research may not be representative of stroke costs and rehabilitation utilization in other states and in the US since healthcare practice patterns and costs vary geographically. In addition, the interpretation of rehabilitation resource use and the assignment of associated costs are challenging.

Due to the lack of availability of clinical information in these claims data, the classification of rehabilitation was based on the inclusion of specific rehabilitation related codes in the ten diagnosis code and four procedure coding columns provided in the data files. Provider billing systems will often allow for a much longer list of these codes when interpreting medical records into billing data in the clinical setting. So it is reasonable to assume that some diagnoses or procedures related to stroke and rehabilitation would not be included in the Medicare data.

## Conclusions

Using a marginal costing approach to estimate healthcare costs due to ischemic stroke is important in the Medicare population compared to using total average costing techniques, in order to accurately attribute healthcare costs to stroke. Indeed, the average total costing approach may be expected to inflate the estimated 2004 SC total cost due to stroke for Medicare patients by $54.4 million, because this approach ascribes expenditures for comorbid conditions to stroke. Healthcare policy makers should be wary of burden of illness estimates that employ average total costs for patients who may be expected to have substantial comorbid disease.

### Consent

This study was deemed non-human subject research. This research did not use personally identifiable patient health information and, therefore did not qualify as Human Subjects Research by the Medical University of South Carolina (MUSC) Institutional Review Board (IRB). Data were de-identified by the non-affiliated data center prior to the time in which the researchers on this project received the data files, therefore this study was deemed a non-human subjects study which required no patient informed consent.

## Abbreviations

SC: South Carolina; AIS: Acute ischemic stroke; SAF: Standard analytic files; ORS: Office of research services; PS: Propensity score; AIC: Akaike information criterion; BIC: Bayesian information criteron.

## Competing interests

The authors declare that they have no competing interests.

## Authors’ contributions

AS conducted all parts of the study as part of a doctoral dissertation project. CE and HB helped to develop the original research question. AK assisted in the development of the theoretical constructs that support the questions. AS, HB, AK, JZ and CE reviewed methods and provided constructive comment during the design and implementation portion of the project. AS and CE drafted the manuscript. All authors read and approved the final manuscript.
